# Reactive oxygen species accelerated glycation for oxygen-independent corneal cross-linking

**DOI:** 10.3389/fbioe.2026.1695384

**Published:** 2026-03-31

**Authors:** Jiashuai Fan, Sinisa Vukelic

**Affiliations:** Department of Mechanical Engineering, Columbia University, New York, NY, United States

**Keywords:** advanced glycation end product, biomechanics, corneal cross-linking, keratoconus, laser therapy

## Abstract

**Background:**

The pentose-initiated Maillard reaction may serve as an oxygen-independent, non-enzymatic crosslinking (CxL) mechanism, but its slow kinetics limit clinical applicability. We hypothesize that glycation-mediated CxL can be accelerated by reactive oxygen species (ROS), potentially enabling its use in the treatment of keratoconus.

**Methods:**

Rabbit corneal buttons were exposed to 20% ribose and irradiated with femtosecond oscillator laser pulses at 450 mW (1,069 nm) and 250 mW (400 nm). In addition to laser-based light sources, a 365 nm UVA lamp at 3 mW/cm^2^ was used in combination with either ribose or riboflavin-5-phosphate to generate comparison and control groups. ROS-Glycation-CxLed corneas were flattened with a glass coverslip to minimize oxygen replenishment. Crosslinking efficacy was assessed using 5 µm micro-indentation to measure the equilibrium modulus and viscoelastic ratio post-treatment.

**Results:**

Corneal tissues subjected to ROS-glycation-CxL exhibited a significant increase in equilibrium modulus. The modulus change in ribose-exposed tissues treated with UVA light was comparable to that observed with the conventional UVA–riboflavin CxL pathway. In contrast, infrared and blue laser-treated samples showed less stiffening, likely due to the limited tissue volume affected by the laser’s focal region. Notably, only the UVA–riboflavin-treated tissues demonstrated a pronounced elastic load-relaxation response.

**Conclusion:**

ROS-Glycation-CxL offers a potential alternative to existing crosslinking techniques, with crucial advantages such as reduced oxygen reliance, light-source agnostic mechanism, and customizable treatment patterns with laser light sources. Further investigation is needed to evaluate the clinical viability of ROS-glycation CxL through optimization of sugar concentration, treatment duration, and assessment of stromal extracellular matrix ultrastructure.

## Introduction

Keratoconus (KCN) is a noninflammatory condition characterized by progressive thinning and weakening of the cornea, which eventually assumes a conical shape. KCN typically begins during adolescence and gradually worsens, leading to irregular astigmatism, myopia, and protrusion (Rabinowitz, 1998). Studies have shown a significant reduction of corneal stromal stiffness in affected individuals (McKay et al., 2019). Collagen crosslinking (CxL) has emerged as an effective treatment for KCN. This procedure introduces non-enzymatic CxL within the stromal extracellular matrix (ECM), primarily composed of collagen fibrils, thereby enhancing its biomechanical strength (Wollensak, 2006).

In a clinical setting, collagen crosslinking for KCN treatment is executed by activation of the riboflavin solution (riboflavin-5-phosphate, or R5P) with ultraviolet A (UVA) light of 365 nm (UVA-Riboflavin-CxL). This non-enzymatic approach to creating CxLs has become a common therapy for treating KCN over the past 2 decades (Meek and Hayes, 2013). Upon UVA light exposure, riboflavin produces reactive oxygen species (ROS) through reaction pathways: Type I and Type II–depending on the availability of oxygen (Meek and Hayes, 2013). Evidence suggests that oxygen and the Type II reaction are essential for effective corneal stiffening (Richoz et al., 2013).

Despite its therapeutic efficacy, UVA-Riboflavin-CxL has several limitations. These include the cytotoxic effects of UVA light on stromal keratocytes and endothelial cells, and the need for epithelial removal to allow riboflavin (R5P) and oxygen to reach the anterior stroma (Evangelista and Hatch, 2018; Dhawan et al., 2011). Epithelial debridement prolongs recovery, increases infection risk, and causes significant discomfort. Consequently, recent efforts have focused on CxL techniques that avoid epithelial removal (trans-epithelial CxL) (Kissner et al., 2010; Wollensak and Iomdina, 2009). Strategies to enhance oxygen and riboflavin delivery include methods such as iontophoresis (I-CxL) (Vinciguerra et al., 2022), direct oxygen supplementation (Mazzotta et al., 2020), laser micro-channeling (Bradford et al., 2020), epithelial flaps (Seiler et al., 2014), as well as chemical enhancers such as benzalkonium chloride (Koppen et al., 2012).

Other efforts to optimize the UVA-Riboflavin-CxL procedure include non-linear activation of riboflavin using femtosecond lasers (Bradford et al., 2016; Bradford et al., 2019; Bradford et al., 2017; Chai et al., 2013), reduction of procedure time (Tomita et al., 2014), and pulsed UVA light irradiation (Sachdev and Sachdev, 2017). Beyond riboflavin, alternative photosensitizers such as 560 nm-activated Rose Bengal with arginine (Wertheime et al., 2020) and 755 nm activated bacteriochlorophyll derivative (WST-11) (Brekelmans et al., 2020; Marcovich et al., 2012; Hayes et al., 2020) offer CxL mechanisms that may facilitate oxygen-independent crosslinking. While their long-term efficacy and safety are still being evaluated, non-riboflavin CxL represents a promising approach for treating KCN without epithelial removal or oxygen dependence.

Another form of non-enzymatic collagen crosslinking is mediated by the Advanced Glycation End product (AGE) *via* the Maillard reaction. In the cornea, glycation occurs naturally with age and is accelerated in diabetes mellitus (DM), where chronic hyperglycemia leads to increased non-enzymatic crosslinking and, consequently, increased corneal stiffness (Kaji et al., 2000; Sady et al., 1995). Glycation-mediated CxL has received limited attention in KCN treatment due to the slow progression of the process under physiological conditions. However, it is possible that these CxLs in individuals with DM may help protect the cornea from KCN development and progression. Although glycation is inherently slow under physiological conditions, it is hypothesized that UV light accelerates the process by generating Reactive Oxygen Species (ROS), which act as catalysts in the glycation-based CxL (Ohan et al., 2002). Other methods for generating ROS have been explored; for example, our group has previously used pulsed femtosecond oscillators to induce low-density plasma, which generates ROS from interstitial water molecules (Wang et al., 2018). Furthermore, smaller open-ring sugars, such as ribose, have been shown to facilitate the glycation-mediated CxL even in the absence of oxygen (Litchfield et al., 1999; D'Oria et al., 2021; Spoerl and Seiler, 1999), suggesting a potential oxygen-independent CxL method. This approach may complement the recent advances in transepithelial UVA-Riboflavin-CxL.

We hypothesize that ROS-Glycation-CxL can enhance the biomechanical properties of the cornea within a clinically relevant timeframe. This study has two aims: first, to investigate the effectiveness of ROS-accelerated, ribose-mediated corneal CxL under conditions of restricted oxygen access to the anterior cornea; and second, to assess whether this CxL method can be achieved using different ROS generation techniques. If effective, the proposed treatment could serve as a robust therapeutic option to arrest KCN progression, and may complement or provide an alternative to the current CxL practices.

## Methods

### Tissue preparation

A total of 12 pairs of fresh rabbit eyes (24 corneas) without visible corneal abnormalities were obtained from a local live poultry vendor (La Granja Live Poultry Corporation, NY, United States of America) within 1-h post-sacrifice. Each eye was enucleated, and its epithelium was carefully removed with a surgical scalpel.

**FIGURE 1:**
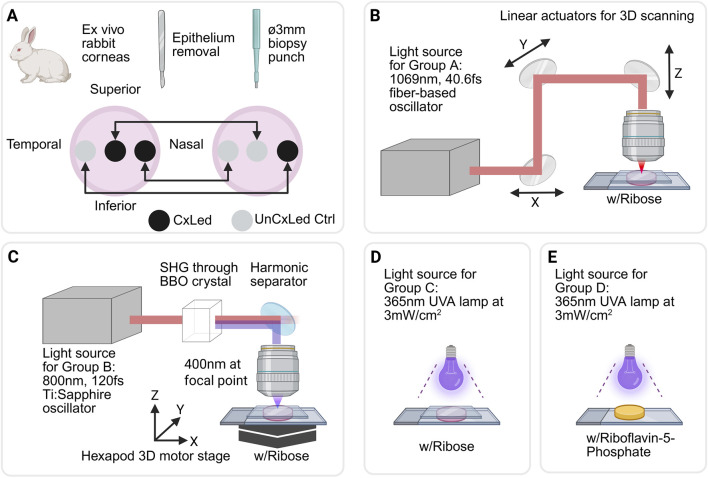
**(A)** Representative corneal sample selection and preparation for laser treatment and mechanical testing. Crosslinking treatments for rabbit corneal samples exposed to 20% ribose *via* **(B)** 1069 nm fiber-based oscillator pulses, **(C)** 400 nm oscillator pulses generated from SHG with an 800 nm Ti:Sapphire laser source, **(D)** a 365 nm UVA lamp, **(E)** Corneal samples crosslinked with R5P and UVA light as positive controls. The figures are made with BioRender.

Corneal buttons (Ø 3 mm) were extracted from both left (OS) and right (OD) eyes using a biopsy punch. The typical extraction sites for the biopsy punch were apical, nasal, temporal, inferior, and superior regions (Figure 1A). To prevent bias from spatial differences in modulus, the locations of the biopsy punches were kept symmetrical between the OS and OD eyes (Singh et al., 2017). For each animal, at least one corneal button from both the OS and OD eyes was selected for crosslinking, to avoid inter-corneal sample bias. Each symmetrical pair consisted of one button subjected to crosslinking (either UVA-Riboflavin-CxL or ROS-Glycation-CxL), and a paired control, treated identically except for light exposure. The UVA-Riboflavin-CxL served as a positive control.

All corneal buttons assigned to groups 1A, 1B and 1C were incubated in 20% ribose (Sigma-Aldrich Inc., United States of America) in phosphate-buffered saline (PBS, HyClone/Cytiva, United States of America) for 1 h; samples assigned to group 1D were prepared for UVA-Riboflavin-CxL (n = 3 rabbits or six corneas each group). To minimize oxygen replenishment, the anterior surfaces of all buttons were pressed with a glass coverslip in Groups 1A, 1B and 1C. Subgroups 1A and 1B were irradiated with femtosecond lasers at 450 mW average power with 1069 nm wavelength, and 250 mW average power with 400 nm wavelength, respectively (Figures 1B,C). Subgroup 1C was exposed to a 365 nm UVA lamp for 1 h, with treated buttons receiving an average of 3 mW/cm^2^ (Figure 1D). Subgroup 1D underwent crosslinking following the well-established Dresden protocol for UVA-Riboflavin-CxL, using the 0.1% riboflavin-5-phosphate in 20% dextran 500 (Sigma-Aldrich Inc., United States of America) and served as the positive control (Figure 1E).

### Treatment

Three different light sources were used to accelerate glycation-mediated CxL: a UV lamp, a femtosecond laser with a central wavelength of 1,069 nm, and a femtosecond laser with a central wavelength of 400 nm.

For glycation acceleration using 1069 nm femtosecond laser, a fiber oscillator (Fidelity II, Coherent, Santa Clara, CA) with a 40.6 femtosecond pulse duration was used. The laser was focused through a Zeiss Plan-NeoFluar ×40 objective yielding 450 mW average power with an effective numerical aperture of 0.146. The objective was mounted onto a 3-axis motorized system with linear actuators (Z825B and PT1, Thorlabs, Newton, NJ) and was moved at a speed of 2.6 mm/s with a maximum acceleration of 4 mm/s^2^ (Figure 1B). Rasterizing in-plane in a zigzag pattern with a 25 μm increments, the laser traced a 3 mm diameter disc parallel to the corneal surface. The treatment pattern was repeated at varying depths from the corneal surface to create a series of ten ‘treatment planes.’

For the 400 nm femtosecond treatment, a tunable Ti:Sapphire oscillator (Chameleon Ultra II, Coherent, Santa Clara, CA) with a 120-femtosecond pulse duration was used. The out-of-cavity laser beam, originally centered at 800 nm and 1.2 mm in diameter, was expanded to 3 mm using a beam expander (BE-02-05X, Thorlabs, Newton, NJ) and then focused into a Beta Barium Borate (BBO) crystal (THG800(I)-A05-100fs, Newlight Photonics, Toronto, Canada) for Type I second harmonic generation to produce 400 nm wavelength beam. A harmonic separator (HS10-R400/T800, Newlight Photonics, Toronto, Canada) filtered the 400 nm beam from the residual 800 nm light. The laser was applied to corneal samples using the same zigzag protocol as above, but with the after-objective average power of 250 mW at the effective numerical aperture of 0.366. The rasterization was performed using a 6-axis motorized hexapod stage (H-840.D2A-AXIS_X, Physik Instrumente, United States of America) at 5 mm/s and a maximum acceleration of 60 mm/s^2^ (Figure 1C).

UVA irradiation was performed using a UV lamp that emits 365 nm light (Crystal Industries, United States of America). The distance between the lamp and the sample surface was adjusted to maintain an average irradiance of 3 mW/cm^2^, as measured by a UV light meter (Sper Scientific Arizona, United States of America) (Figures 1D,E).

### Micro-indentation

**FIGURE 2:**
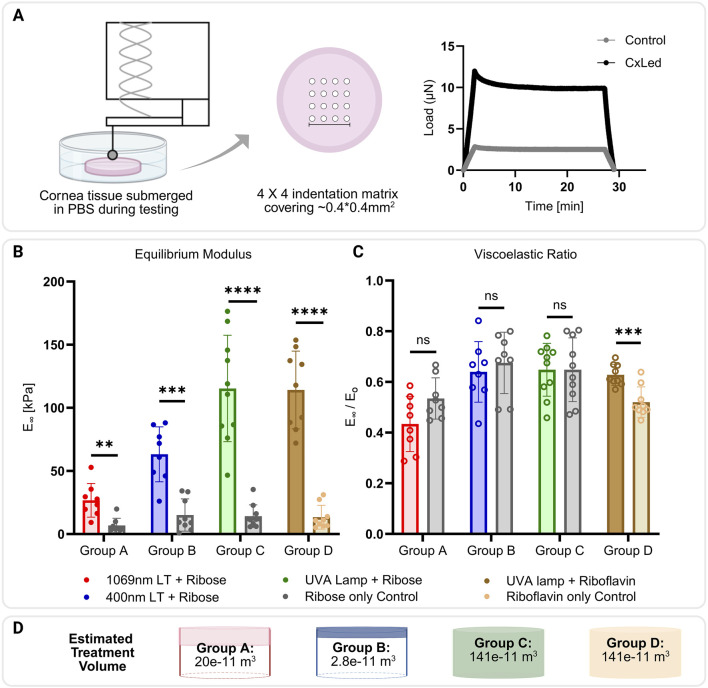
**(A)** Representative schematic of micro-indentation performed on corneal buttons submerged in PBS, and resulting stress-relaxation curves from crosslinked and control samples. **(B)** Statistically significant differences found between average equilibrium modulus of crosslinked and paired control samples for Group1 A, B, C for ROS-Glycation-CxL and D for UVA-Riboflavin-CxL (**p < 0.01; ***p < 0.001; ****p < 0.0001). **(C)** Viscoelastic ratio calculated from instantaneous and equilibrium moduli. Only crosslinked samples in Group 1D had a significantly different time-dependent elastic response than the uncrosslinked control (***p < 0.001). **(D)** Estimated treatment volumes across each group assuming a cylindrical stromal volume with 3 mm diameter and 200 μm depth. The figures are made with BioRender.

Following CxL treatment, the corneal buttons were incubated overnight in PBS at 4 °C to achieve hydration equilibrium. Paired samples were always dissected from symmetrical positions in matched corneas with comparable stromal ECM structures, ensuring optimal biomechanical comparability; three to four pairs of buttons were trephined per rabbit. Indentations performed in any hydration state other than the equilibrium were identified by an ascending, rather than horizontal displacement-controlled stress-relation curve (Figure 2A) indicating a “swelling” surface and were excluded from analysis.

After incubation, the trephined corneal buttons were brought to room temperature and secured to the bottom of a Petri dish with the anterior surface facing up. A displacement-controlled indenter (Piuma, Optics11, Amsterdam, Netherlands) was used to perform micro-indentations on the sample surface submerged in PBS. Each sample was tested at equally spaced contact points (4 × 4 grid) with 100–150 μm spacing, covering approximately a 0.4 × 0.4 mm^2^ area at the center of each corneal button. For Group A, a spherical glass probe with a 34 μm radius and 0.2 N/m stiffness. For Groups B, C, and D stiffer probe (25.5 μm radius, 4.09 N/m stiffness) was necessary due to higher mechanical strength of samples exceeding the measurement range of the softer probe. The indentation process at each contact point was comprised of three steps: loading with a Piezo movement of 5 μm over 2 s, holding (5 s), and unloading (2 s) (Figure 2A).

#### Data analysis

The load-relaxation indentation experiments were analyzed using a viscoelastic framework to obtain the reported equilibrium modulus and viscoelastic ratio, following a fitting algorithm in MATLAB with a nonlinear least-squares solver. Specifically, the material was modeled as a generalized Maxwell solid consisting of a linear spring with 2 Maxwell branches (a linear spring and a dashpot linearly connected). In an isotropic elastic Hertzian contact with spherical indentation on an incompressible material, the load can be expressed as (Mattice et al., 2006; Fodera et al., 2024; Islam et al., 2020):

$$P=\frac{4\sqrt{R}}{3}\frac{E}{\left(1-\upsilon^{2}\right)}h^{1.5}$$

where *R* is the spherical probe radius, h is the displacement, υ is the Poisson’s ratio (0.5 for incompressible solid), E is the elastic modulus, and G is the shear modulus. The relationship between elastic and shear modulus is commonly taken as E=3G .

The elastic Hertzian solution was extended to obtain the generalized Boltzmann integral under displacement control (Mattice et al., 2006):

$$P\left(t\right)=\frac{8\sqrt{R}}{3}\int_{0}^{i}G\left(t-u\right)\left[\frac{d}{du}h^{1.5}\left(u\right)\right]du$$

where $h\left(t\right)=h_{\max}\;at\;t=t_{ramp}$ . The shear relaxation function was assumed to be:

$$G\left(t\right)=C_{0}+C_{1}e^{-t/\tau_{1}}+C_{2}e^{-t/\tau_{2}},$$

and the load-relaxation portion of the experimental load curve can be expressed as:

$$P\left(t\right)=B_{0}+B_{1}e^{-t/\tau_{1}}+B_{2}e^{-t/\tau_{2}},$$

with $\tau_{1,2}$ denoting the viscoelastic relaxation time. An approximate relationship can relate the fitting parameters $B_{0,1,2}$ to material parameters $C_{0,1,2}$ as

$$C_{0}=\frac{B_{0}}{h_{\max}^{1.5}\left(\frac{8\sqrt{R}}{3}\right)},C_{1,2}=\frac{B_{1,2}}{h_{\max}^{1.5}\left(\frac{8\sqrt{R}}{3}\right)\left(RCF_{1,2}\right)},$$

where RCF is the ramp correction factor, describing the experimental ramp time, defined as

$$RCF_{1,2}=\frac{\tau_{1,2}}{t_{ramp}}\left[e^{\frac{t_{ramp}}{\tau_{1,2}}}-1\right].$$

The instantaneous shear modulus $G_{0}$ and long time or equilibrium shear modulus $G_{\infty }$ was expressed as

$$G_{0}=\frac{C_{0}+C_{1}+C_{2}}{2},G_{\infty }=\frac{C_{0}}{2}.$$

The “reduced elastic modulus” adapted in the software is defined as $E_{r}=2G/\left(1-\upsilon \right)=4G$ (Xu et al., 2022). Both instantaneous and equilibrium moduli reported in this study are reduced elastic modulus derived from fitted viscoelastic parameters. Specifically, the instantaneous modulus is $E_{0}=4G_{0},$ and the equilibrium modulus is $E_{\infty }=4G_{\infty }$ . The viscoelastic ratio is taken to be $E_{\infty }/E_{0}$ .

#### Statistical analysis

Mechanical indentation data were analyzed using Prism 10.6.0 (GraphPad Software, CA, United States of America). Because treated and control samples were harvested from spatially corresponding regions of paired rabbit eyes, comparisons of equilibrium modulus and viscoelastic ratio were performed using paired t-tests. Statistical significance was defined at a 95% confidence level (α = 0.05). P-value notation follows conventional formatting: ns (p > 0.05), *p < 0.05, **p < 0.01, ***p < 0.001, ****p < 0.0001.

## Results

The equilibrium modulus significantly increased in all groups after ROS-Glycation-CxL. In contrast, paired controls consistently exhibited average modulus of around 10 kPa. Specifically, crosslinked corneas exhibited equilibrium moduli of 25 kPa for 1069 nm laser treatment (Group 1A, p < 0.01), 60 kPa after 400 nm laser treatment (Group 1B, p < 0.001), and 100 kPa after 365 nm UVA lamp treatment (Group 1C, p < 0.0001). The positive control group, treated with the UVA-Riboflavin-CxL protocol, also demonstrated a significant increase in equilibrium modulus to 100 kPa (p < 0.0001) (Figure 2B).

No significant changes in the time-dependent viscoelastic ratio were observed among ROS-Glycation-CxL samples or their non-CxLed paired controls, irrespective of the light source used. However, corneas treated with the UVA-Riboflavin-CxL protocol exhibited a more elastic response compared to their respective paired controls (p < 0.001) (Figure 2C).

## Discussion

In this study, we have explored the synergistic effects of ROS and non-enzymatic glycation using ribose on crosslinking in rabbit corneal stroma. The cornea, a load-bearing ocular tissue, accounts for approximately 80% of the eyes’ refractive power. Its biomechanical integrity is essential for maintaining the appropriate curvature necessary for light refraction onto the retina under intraocular pressure (Hayes et al., 2013). Conditions such as KCN reduce the mechanical strength of the corneal stroma. This prompted the clinical adoption of UVA-Riboflavin crosslinking to retard disease progression (Gaster et al., 2013). Here, we investigated non-enzymatic glycation as an alternative crosslinking strategy to modify the mechanical properties of the cornea. Given that pentose-mediated glycation is largely oxygen-independent (Litchfield et al., 1999), we minimized oxygen exposure during the crosslinking by placing a coverslip against the anterior surface, and surrounded the corneal button with the ribose PBS solution. A high concentration of ribose (20%) in PBS solution ensured an adequate supply of sugar molecules.

While glycation itself can induce crosslinks under physiological conditions, this process typically requires prolonged incubation periods, and sugars such as ribose and glucose are relatively ineffective crosslinkers (Spoerl and Seiler, 1999; Hanna Tręb et al., 2018). Previous studies have shown that oxidative stress and the introduction of photo-generated ROS can catalyze the crosslinking by facilitating AGE-mediated products (Moldogazieva et al., 2019). In this study, we combined ribose solution with various light sources to accelerate AGE-mediated crosslinking (CxL) in the corneal stroma. We used three light sources to catalyze the process: an infrared (IR) femtosecond laser, a UVA lamp, and a UV femtosecond laser. Our previous studies demonstrated that the IR femtosecond laser operating at nano-joule pulse energy creates low-density plasma in the interior of the corneal stroma, ionizing and dissociating interstitial water to produce ROS (Wang et al., 2018). The 365 nm UV-A lamp, commonly used in traditional corneal crosslinking protocols, generates free radicals within collagen (Ohan et al., 2002; Moldogazieva et al., 2019). Lastly, we utilized a Type I SHG mechanism to convert an 800 nm Ti: Sapphire femtosecond oscillator beam to 400 nm, allowing us to study ROS generation by UVA/blue light ultrafast laser (Kulatilaka et al., 2014).

To evaluate the effects of treatment, we chose micro-indentation with a 5 μm indentation depth. This method enables assessment of tissue mechanical properties at micrometer to nanometer scales on the surface by varying the size and shape of the indenter. Such flexibility is particularly advantageous for characterizing mechanical alterations induced by photochemical collagen CxL, as the effects of photochemical crosslinking are most apparent at the treatment surface due to light absorption and scattering. Specifically, for a tightly focused laser beam with a focal volume of less than 50 μm^3^, nanoindentation provides greater spatial sensitivity than large-scale tests, such as uniaxial tensile tests (Fodera et al., 2024; Komninou et al., 2024).

Measurements of the equilibrium modulus and the time-dependent viscoelastic ratio were conducted at multiple points within a prescribed indentation matrix following ROS-Glycation-CxL treatment. For all three ROS generation methods for accelerating ribose-based glycation, the average equilibrium modulus of crosslinked tissue was significantly higher than that of the ribose-exposed-only, coverslip-pressed controls. Among these methods, the 1,069 nm laser irradiation produced the least amount of stiffening, yielding a modulus of 26.7 kPa, approximately 3.8-fold the control value. In contrast, 400 nm laser irradiation increased the modulus to about 63.2 kPa, or about 4.6 times the control. We hypothesize that the higher photon energy at 400 nm led to a more efficient ROS generation and, consequently, greater tissue stiffening. Higher energy UV/blue wavelengths are known to be more effective for collagen modification in the corneal tissues than IR or near-IR wavelengths (Hovhannisyan et al., 2010; Hovhannisyan et al., 2008; Huang et al., 2022), which is also observed in the autofluorescence and SHG signal of Group C samples post-treatment (Supplementary Figure 1). Notably, treating with a 365 nm UVA lamp for 1 hour produced a modulus of 115.3 kPa, approximately 8.2 times the paired control. This magnitude of equilibrium modulus change is on par with that observed with UVA-Riboflavin-CxL, establishing the strong stiffening potential of the ROS-Glycation-CxL.

The substantial stiffening observed in samples treated with UVA lamp in Groups C and D may also be attributed to the larger volume of crosslinked tissue. Laser-mediated treatments were limited to the anterior 200 μm to match the Dresden protocol, which has a demarcation line around the 200 μm from the anterior surface. While both laser-catalyzed glycation CxL and UV-Riboflavin-CxL penetrate to similar depths, the total tissue volume affected by laser is significantly smaller. In laser-assisted CxL, ROS are primarily generated within and in the vicinity of the focal volume, whereas the UVA lamp affects the entirety of the exposed stroma. To illustrate this difference, consider a cylindrical stromal volume with a diameter of 3 mm and a depth of 200 μm. The UVA lamp treats the entire volume, which is approximately 1.41 × 10^−9^ m^3^. In contrast, the effective treatment volumes of IR and UV lasers, based on the size of the multiphoton focal volume and our current raster scan regime (Zipfel et al., 2003), are only 20.0 × 10^−11^ m^3^ and 2.80 × 10^−11^ m^3^, respectively. Thus, laser treatments cover only about 2%–14% of the total stromal volume affected by the UVA lamp (Figure 2D). Although a clear demarcation zone in UV lamp-catalyzed, glycation-mediated CxL was not observed, and thus we cannot tell with certainty the extent of the crosslinked region, the stiffening achieved is similar to that of the positive controls. This indicates that the CxL efficacy of two crosslinking modalities are similar when mediated by a UVA lamp.

None of the ROS-Glycation-CxL treatments produced a statistically significant difference in the time-dependent viscoelastic ratio compared to the paired controls. In contrast, the UVA-Riboflavin-CxL treatment elicited a more elastic tissue response, characterized by a reduced difference between the initial contact force and equilibrium force observed in the load-relaxation curve. This disparity between the two crosslinking mechanisms might be attributed to distinct alterations and crosslinking sites within the stromal ultrastructure, which determines how the collagen lamellae and fibrillar structures crimp, rotate, and stretch under loading conditions during the micro-indentation testing (Bell et al., 2018; Meek and Quantock, 2001).

Another important future direction is to investigate the specific crosslinks formed in ROS glycation crosslinking, as these crosslinks determine the biomechanical behavior of the treated tissue (Depalle et al., 2015; Gautieri et al., 2014). Toward this goal, collagen fluorescence can be used as an indicator of crosslink density and crosslink type (Chai et al., 2013; Raub et al., 2007). We briefly surveyed the autofluorescence signal of crosslinked tissue in this study (Supplementary Figure 1). When captured in an emission window of 475 nm–650 nm, crosslinks formed in ROS-Glycation-CxL had significantly less autofluorescence intensity than UVA-Riboflavin-CxL, which produces flavoproteins with an emission peak at 550 nm (Supplementary Figure 1). However, this detection window may not have covered autofluorescence from collagen-AGE crosslinks. To better understand the molecular types and nature of crosslinks in ROS-Glycation-CxL, future investigation is needed to scan a broader fluorescent emission range related to AGEs (370 nm–460 nm), covering emission peaks from relevant AGEs such as pentosidine, vesperlysine, crossline, fluorolink, and argyrimidine (Séro et al., 2013; Perrone et al., 2020; Rovati and Docchio, 2004).

Overall, ROS-Glycation-CxL demonstrates considerable potential as an alternative to traditional UVA-Riboflavin-CxL, as it may function independently of oxygen and is agnostic to the photochemical source (Mun et al., 2014; Nawaz et al., 2015). Additionally, the 400 nm laser treatment effectively enhanced corneal mechanical properties and holds promise for efficient ROS generation at relatively low power, potentially enabling spatially precise and complex CxL patterns. Further investigation is warranted to understand the changes ROS-Glycation-CxL induced in the ultrastructure of the stromal extracellular matrix. To improve the practicality of ROS-Glycation-CxL, future research will explore lower sugar concentrations and shorter, repeated UVA lamp exposure to facilitate clinical translation. Additionally, other simpler carbohydrates such as tetrose and glyceraldehyde, and smaller dicarbonyls such as glyoxal and methylglyoxal (Litchfield et al., 1999), are also worth surveying in the ROS-Glycation-CxL as potentially more capable, oxygen-independent glycating agents.

## Data Availability

The original contributions presented in the study are included in the article/Supplementary Material, further inquiries can be directed to the corresponding author.
