# Supplementary material for: Reactive oxygen species accelerated glycation for oxygen-independent corneal cross-linking
Source: Front Bioeng Biotechnol. 2026 Mar 31;14:1695384. doi: 10.3389/fbioe.2026.1695384 (PMC13076249; doi:10.3389/fbioe.2026.1695384)
Supplement: Supplementary file 1 [file Supplementaryfile1.docx]

**Reactive Oxygen Species Accelerated Glycation for Oxygen-Independent Corneal Cross-Linking**

Jiashuai Fan, Sinisa Vukelic

Department of Mechanical Engineering, Columbia University, New York, NY10027

**Supplemental Materials: Multiphoton Imaging**

**
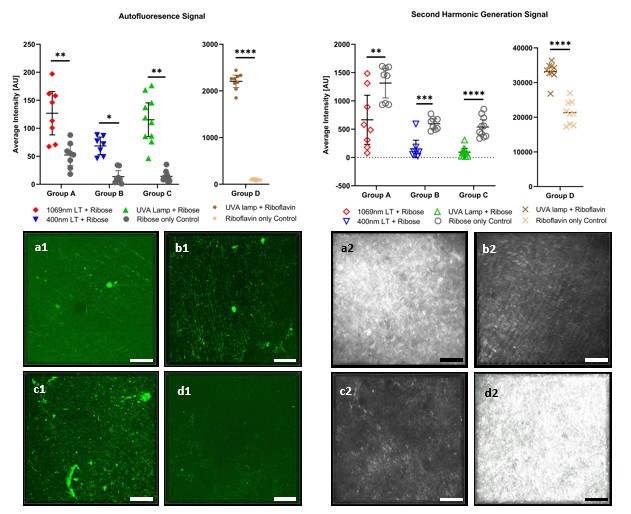
**

**Supplemental Figure 1**. Autofluorescence and second harmonic generation (SHG) signal intensities from multiphoton imaging for treatment Groups A, B, C, D. Statistical significance was found for all treated and control pairs. Representative image stacks (612 *612*100μm) are shown in the XY orientation at a) 1069nm laser-treated corneas, b) 400nm laser-treated corneas, c) UVA lamp-treated corneas, and d) ribose only controls for Groups A, B, C, D. Scale bar = 100μm.

**Methods**

The autofluorescence and second harmonic generation (SHG) signals of the corneal tissues were investigated to reveal the presence of CxLs and structural changes in collagenous ECM after hydration equilibrium in PBS on the same night of individual treatment days. Imaging was performed with a Nikon TI Eclipse inverted microscope using a Ti:Sapphire laser Chameleon Ultra II at 860nm as the light source and a Nikon Apo LWD 20x objective for focusing at the Columbia University Herbert Irving Comprehensive Cancer Center (HICCC) Confocal and Specialized Microscopy. The emission filter was set at the FITC channel (475-650nm, peak 525nm) for autofluorescence signal collection and at the DAPI channel (400- 600nm, peak at 457nm) for SHG signal collection. The XY scanning area for each sample was 635μm by 635μm, and Z scanning was 100μm deep with 10μm increments (stack of 11 images

per sample). The summation of the average grey value processed in Image J at each layer was

used as the representative signal strength for each sample.

**Results**

The autofluorescence signal intensity of the anterior 100μm of corneal tissues increased significantly for all three ROS-Glycation-CxL groups with 1069nm laser treatment in Group A (p<0.01), 400nm laser treatment in Group B (p<0.05), and 365nm UVA lamp treatment in Group C (p<0.01) compared to the respective paired controls. However, the observed difference is small in magnitude when compared against the autofluorescence increase observed in corneal buttons subjected to the UVA-Riboflavin-CxL protocol (p<0.0001). An apparent signal increase can be observed in laser scanning tracks for 400nm laser treatment but not for 1069nm laser treatment (**Supplemental Figure 1**).

The second harmonic generation signal intensity of the anterior 100μm corneal tissues decreased significantly for all three ROS-Glycation-CxL groups with 1069nm laser treatment in GroupA (p<0.01), 400nm laser treatment in Group B (p<0.001), and 365nm UVA lamp treatment in Group C (p<0.0001) compared to the ribose only control. Conversely, UVA- Riboflavin-CxL protocol CxLed corneas showed increased SHG signal compared to R5P exposed paired control. A clear decrease in signals can be observed in laser scanning tracks for 400nm laser treatment but not for 1069nm laser treatment (**Supplemental Figure 1**).

**Discussion**

Collagen autofluorescence has been used as another indicator for the formation of crosslinking [1]. It has been shown that for UVA-Riboflavin-CxL, the signal intensity of collagen autofluorescence positively correlates with crosslinking efficiency and biomechanical enhancement [2]. Additionally, after infrared femtosecond laser ROS-induced crosslinking, collagen autofluorescence signal has also been reported to increase in the raster-scanned region [3]. For all three ROS generation methods, after ROS-Glycation-CxL, the autofluorescence signal intensity, as the sum of grey values in the imaged volume at the anterior 100μm stroma, slightly increased compared to the paired control. For the positive control Group D, the autofluorescence signal of the UVA-Riboflavin-CxL protocol crosslinked group has also increased as expected. However, the magnitude of autofluorescence change is dramatically different. We suspect that the emission window used in this study, between 475nm to 650nm, covers the flavoproteins (emission peak at 550nm) that originate from the riboflavin-5-phosphate induced crosslinking but does not cover the pentosidine-type crosslinking (emission peak at 380nm) that might from in glycation process [4].

The second harmonic generation signal captured in corneal stroma comes from the interaction of light with non-centrosymmetric structures in collagen type I. Due to this underlying intrinsic origin, the signal is highly sensitive to collagen fibril and fiber ultrastructure, which is subject to change after CxL [5]. We observed a statistically significant increase in the summary of grey value at the most anterior 100μm of UVA-Riboflavin-CxLed cornea tissues. In contrast, we observed statistically significant decreases of SHG signal in treated tissues after ROS-Glycation-CxLed for all three light sources. Interestingly, for the 365nm UVA-lamp irradiated and 400nm laser-treated groups with higher stiffening, the SHG signal decreased more than the 1069nm laser-treated group with less mechanical strength modification. We suspect the different trends observed in SHG signal alterations after CxL are partially due to how ROS-Glycation changed the original collagenous ECM structure in a different mechanism than the UVA-Riboflavin. It is also possible that compared to Riboflavin-5-Phosphate, Ribose might not have had equally effective protection against ultraviolet light, and there was potentially some fibrillar scission or unraveling due to highly concentrated exposure to ROS.

We observed laser scanning patterns in the 400nm group but not in the 1069nm group (**Supplemental Figure 1 c1 and c2**). There might be two reasons behind this. First, though the average power at 1069nm (450mW) is higher than the power at 400nm (100mW), due to out-of-cavity beam expansion required for the SHG crystal, the 400nm beam path may have had a much higher effective numerical aperture, resulting in a much more focused beam. Second, it has been shown that high UV, blue wavelength is much more effective at collagen manipulation in the corneal tissues compared to the IR or near-IR wavelength. This trend of increased autofluorescence and decreased SHG signal coincidently agrees with other studies using femtosecond oscillator's manipulation with collagenous tissue in similar studies [6-8].

1. Raub, C.B., et al., *Noninvasive assessment of collagen gel microstructure and mechanics using multiphoton microscopy.* Biophys J, 2007. **92**(6): p. 2212–22.

2. Chai, D., et al., *Quantitative Assessment of UVA-Riboflavin Corneal Cross-Linking Using Nonlinear Optical Microscopy.* Investigative Ophthalmology & Visual Science, 2011. **52**(7): p. 4231–4238.

3. Wang, C., et al., *Femtosecond laser crosslinking of the cornea for non-invasive vision correction.* Nature Photonics, 2018. **12**(7): p. 416–422.

4. Rovati, L. and F. Docchio, *Autofluorescence methods in ophthalmology.* J Biomed Opt, 2004. **9**(1): p. 9–21.

5. Aghigh, A., et al., *Second harmonic generation microscopy: a powerful tool for bio-imaging.* Biophys Rev, 2023. **15**(1): p. 43–70.

6. Hovhannisyan, V., et al., *Photophysical mechanisms of collagen modification by 80 MHz femtosecond laser.* Opt Express, 2010. **18**(23): p. 24037–47.

7. Hovhannisyan, V., et al., *Dynamics of femtosecond laser photo-modification of collagen fibers.* Opt Express, 2008. **16**(11): p. 7958–68.

8. Huang, R., et al., *Blue-LIRIC in the rabbit cornea: efficacy, tissue effects, and repetition rate scaling.* Biomed Opt Express, 2022. **13**(4): p. 2346–2363.
